# Snap Your Fingers! An ERP/sLORETA Study Investigating Implicit Processing of Self- vs. Other-Related Movement Sounds Using the Passive Oddball Paradigm

**DOI:** 10.3389/fnhum.2016.00465

**Published:** 2016-10-10

**Authors:** Christoph Justen, Cornelia Herbert

**Affiliations:** ^1^University of TübingenTübingen, Germany; ^2^Applied Emotion and Motivation Research, Institute of Psychology and Education, University of UlmUlm, Germany

**Keywords:** finger snapping sounds, EEG, N2a/MMN, P3, source localization

## Abstract

So far, neurophysiological studies have investigated implicit and explicit self-related processing particularly for self-related stimuli such as the own face or name. The present study extends previous research to the implicit processing of self-related movement sounds and explores their spatio-temporal dynamics. Event-related potentials (ERPs) were assessed while participants (*N* = 12 healthy subjects) listened passively to previously recorded self- and other-related finger snapping sounds, presented either as deviants or standards during an oddball paradigm. Passive listening to low (500 Hz) and high (1000 Hz) pure tones served as additional control. For self- vs. other-related finger snapping sounds, analysis of ERPs revealed significant differences in the time windows of the N2a/MMN and P3. An subsequent source localization analysis with standardized low-resolution brain electromagnetic tomography (sLORETA) revealed increased cortical activation in distinct motor areas such as the supplementary motor area (SMA) in the N2a/mismatch negativity (MMN) as well as the P3 time window during processing of self- and other-related finger snapping sounds. In contrast, brain regions associated with self-related processing [e.g., right anterior/posterior cingulate cortex (ACC/PPC)] as well as the right inferior parietal lobule (IPL) showed increased activation particularly during processing of self- vs. other-related finger snapping sounds in the time windows of the N2a/MMN (ACC/PCC) or the P3 (IPL). None of these brain regions showed enhanced activation while listening passively to low (500 Hz) and high (1000 Hz) pure tones. Taken together, the current results indicate (1) a specific role of motor regions such as SMA during auditory processing of movement-related information, regardless of whether this information is self- or other-related, (2) activation of neural sources such as the ACC/PCC and the IPL during implicit processing of self-related movement stimuli, and (3) their differential temporal activation during deviance (N2a/MMN – ACC/PCC) and target detection (P3 – IPL) of self- vs. other-related movement sounds.

## Introduction

We are exposed to a diversity of different sounds when interacting with our social environment (e.g., voices, music beats, car noises, ring tones of mobile phones and alarm clocks, the sounds of coffee machines, or footsteps). Crucially, each of these sounds may be matched to a unique and characteristic sound source in our acoustic environment. One of the most salient and special types of sounds relevant to our self are those produced by ourselves (e.g., own voice or movement sounds produced during walking, finger tapping, or finger snapping) as well as those sounds produced by other persons in our close surrounding (e.g., other person’s voice, footsteps, finger snapping sounds, etc.). Hence, to successfully interact with our social environment, we need to identify whether a perceived sound pattern is behaviorally relevant as well as belonging to us or another person (e.g., one’s own vs. another person’s voice) and if it is produced by our own or another one’s actions and movements, such as one’s own vs. other people’s footsteps, hand clapping, or finger snapping sounds (Gillihan and Farah, 2005). A perceived stimulus is assigned as being behaviorally relevant, only if this stimulus has a clear self-reference (Damasio and Meyer, 2009). As such, salience is defined as “the top–down intentionally driven behavioral relevance of the stimulus” (c.f. Joos et al., 2014, p. 151).

In fact, the distinction between the self and the external environment is an essential feature of our psychological life (James, 1890/1950; Gillihan and Farah, 2005). Not surprisingly, though, the distinction between “self” and “other” as well as self-related processing, in general, have attracted the attention of cognitive neuroscience (e.g., see Christoff et al., 2011). The temporal and spatial dynamics involved in implicit and explicit self-related processing of stimuli such as one’s own face or the subject’s own name, have been investigated in a plethora of previous studies (e.g., see Nakamura et al., 2001; Kaplan et al., 2008; Tacikowski and Nowicka, 2010; Tacikowski et al., 2014) as well as in meta-analytic research (Northoff et al., 2006). The findings of this research suggest that particular regions in the brain such as the cortical midline structures (CMS) are involved in the processing of self-related stimuli. This has also been supported by a number of recent studies using less familiar self-related stimuli than the subject’s own name or face such as personal and possessive pronouns (e.g., see Sui et al., 2006; Esslen et al., 2008; Zhou et al., 2010; Herbert et al., 2011) or the subject’s own belongings such as one’s own shoes, umbrella or mobile phone (Miyakoshi et al., 2007; Roye et al., 2010).

On the neurophysiological level, the results of these studies investigating modulation of event-related brain potentials revealed facilitated processing of self-related stimuli as early as in the N2/P2 window for highly salient and familiar stimuli such as the subject’s own name or face (e.g., see Caharel et al., 2002). Similar or temporally even earlier event-related potential (ERP) differences, e.g., in the P1 time-window, have been reported, for instance, during visual or acoustic presentation of self- vs. other-related pronouns (Sui et al., 2006; Blume and Herbert, 2014; Herbert et al., 2016) or when individuals were presented with familiar and unfamiliar own vs. another subject’s belongings (Miyakoshi et al., 2007). Taken together, these studies including those using less familiar and less salient self-related stimuli than the subject’s own name or face suggest that our brain rapidly and effortlessly discriminates between self- and other-related stimuli even if we are not explicitly instructed to do so. Thus, this preferential processing of self-related stimuli seems to hold true even during mere stimulus exposure when no explicit identification of the stimuli as self-related is required. However, despite the abundant evidence summarized above, it is still unclear whether preferential processing of self-related stimuli also holds true for sensory information produced by own vs. another person’s movements (e.g., the acoustic sounds produced by our own or another person’s finger snapping, tapping, footsteps, and so forth) and if so, what the neural sources underlying this kind of implicit self-recognition would be.

In everyday life, we often produce hand or finger movements associated with a characteristic sound pattern, e.g., hand clapping or finger snapping. Regarding the sound of a finger snap, it is not only tightly related to oneself, in addition, it is also important for social behavior as finger snapping is often expressed in real life situations when we are interacting with other people (e.g., while singing and/or making music in a band) or in order to capture other people’s attention (e.g., students trying to get their teacher’s attention). Self-generated movement sounds are dynamic stimuli that are characterized by distinctive and possibly unique sound features (MacPherson et al., 2009). Hence, each person’s movement (e.g., a finger snap) should generate a characteristic and perceivable sound pattern that might be distinguished from the sound pattern originating from movement-related information produced by another person, even if the movement is not executed during stimulus presentation (e.g., during the temporally separated perception of previously recorded movement sounds).

Support for this assumption comes from neurophysiological research in monkeys and from research on mirror neurons and action observation. In an animal study, Kohler et al. (2002) could demonstrate an increase in firing rates in ventral premotor regions (F5 mirror neurons) while monkeys were simply listening to the sounds of actions without performing the action. Firing rates were increased only for movement-related sounds (and, for instance, not while monkeys were listening to interesting sounds or own or another monkey’s vocalizations). However, the firing rate of these neurons did not differ between sounds that were recorded from the monkey’s own or another monkey’s movement. Research on human mirror neurons, action observation and action imitation supports these results suggesting that specific brain areas in the inferior frontal gyrus (IFG) and ventral premotor cortex, belonging to the brain’s mirror neuron systems, show enhanced activation when performing, imitating or observing hand, foot, or finger movements and also when only listening to the sounds of body movements. Thus, our brain seems to be equipped with special multimodal mirror neurons that code multimodal information about movements even in the absence of these movements.

Despite this evidence, so far, only a few neurophysiological studies with human participants have investigated how and where sensory information produced by one’s own body movements is discriminated in the brain from sensory information produced by another person’s movements, when the movement is not executed during stimulus presentation (e.g., during the temporally separated perception of previously recorded movement sounds).

In a recent study, Justen et al. (2014) asked subjects to evaluate previously recorded movement-related stimuli regarding their self- or other-relatedness. Relatively complex movement-related stimuli consisting of long-jump sounds that were recorded across several seconds were chosen and then presented acoustically to participants who were asked to recognize the sounds supposed to be related to their own previously recorded long jumps while the electroencephalogram (EEG) was recorded. A subsequent analysis using standardized low-resolution brain electromagnetic tomography (sLORETA) localized major neural sources in the right anterior cingulate cortex/medial prefrontal cortex [ACC/MPFC; Brodmann area (BA) 32] and in the right temporoparietal junction (TPJ; BA 40). These cortical regions are frequently associated with self-related processing (e.g., see Qin and Northoff, 2011). However, no specific activation in the IFG or premotor cortical regions was observed. Due to the complexity of the stimuli, no analysis of the time course of stimulus processing was included in the EEG study. Thus, it leaves open the question, at which stages of information processing sounds related to the listener’s own long jumps could be earliest discriminated from sounds related to the long jumps belonging to another participant and importantly, whether self-other discrimination would have occurred if sound discrimination had been based on implicit self-related processing. Implicit self-related processing is thought to be mainly related to automatic processing occurring below the level of consciousness and may, therefore, better reflect the operation of self-related processing in everyday life (e.g., in the domain of self-voice recognition, see Graux et al., 2013, 2015). For example, one usually does not explicitly reflect about whether he/she is snipping fingers to a musical rhythm. Alternatively, one is guided by his/her own implicit self-knowledge to make an automatic, adaptive and suitable (behavioral) response.

Event-related potential studies offer the possibility to investigate self-related processing in the temporal domain, even during passive stimulus presentation. Moreover, ERP components can be seen as important neurophysiological indicators of fundamental stages of stimulus processing, spanning the whole processing range from the simple decoding of stimulus features up to conscious perception (e.g., see Kotchoubey, 2006). Using the advantage of ERPs, Hauk et al. (2006) examined the time course of the processing of natural and artificial human finger snapping sounds that were embedded in a passive oddball paradigm. Natural movement-related sounds produced larger mismatch responses in the ERPs than artificially produced control stimuli. This was indexed by the so-called mismatch negativity (MMN) already 100 ms after stimulus onset. Source localization results revealed activation for natural finger snapping sounds in the left primary motor cortex (M1; BA 6) in line with the view mentioned above, that sensorimotor regions, premotor and motor cortex, in particular, are significantly involved in recognizing movement-related body signals, even if the movement itself is not executed. Unfortunately, Hauk et al. (2006) only used natural and artificial movement-related sounds with no clear self-relatedness. Hence, it remains open (1) when and where during stimulus processing the human brain distinguishes self-related from non-self-related auditory movement-related information when the movement itself is not executed during stimulus presentation and crucially (2) whether the observed brain activity patterns would be specific for the perception of sounds related to one’s “own” vs. “another” person’s movements or (3) would also occur during discrimination of sounds that are movement-unrelated. To shed light on these questions, the present study uses a passive oddball paradigm with (1) movement-unrelated pure tones devoid of any personal reference and (2) sounds previously recorded from one’s own and other-related finger snapping sounds and investigates modulation of ERPs elicited during passive listening to these stimuli in combination with sLORETA.

To study ERPs and their modulation by different kinds of stimuli (pure tones vs. self- and other-related snapping sounds) in the present study, the well-established and robust auditory oddball paradigm was used (Squires et al., 1975). During the oddball paradigm, stimuli are typically presented as rare (or deviant) stimuli (e.g., pure tones or natural sounds) among frequent (or standard) stimuli. Deviant differ from standard stimuli in at least one perceptual dimension (for instance, a difference in frequency, pitch or loudness; Rosburg, 2003). According to the literature, both standard and deviant stimuli elicit the N1 component (with highest amplitudes for deviant stimuli; Luck, 2005) which is thought to be a neurophysiological correlate of selective attention (Luck and Hillyard, 1994) and working memory (Hillyard et al., 1973). For acoustic stimuli such as pure tones, the main neural generators of the N1 can be found in the primary and secondary auditory cortices (A1 and A2; BAs 41/42) in the superior temporal gyri (STG)/Heschl’s gyri and adjacent to the planum temporale (PT; Zouridakis et al., 1998; Godey et al., 2001). Besides the N1, deviant stimuli have been found to elicit the N2 and the P3 component (for an extensive introduction, see Luck, 2005). The N2 component is a negative deflection in the ERP waveform, primarily with a scalp topography over anterior electrode sites. Given the existing literature, the N2 can be subdivided into three distinct components: the auditory N2a or mismatch negativity (MMN), the N2b, and the N2c component, respectively (Patel and Azzam, 2005)^1^. The MMN is associated with auditory deviance detection (e.g., see Grimm and Escera, 2012). It is computed by subtracting the averaged ERPs elicited by standard stimuli from those in response to deviant stimuli. As the N2a/MMN is peaking between 100 and 250 ms after stimulus onset (Garrido et al., 2009), it may even overlap with the aforementioned N1 (e.g., see Campbell et al., 2007) that usually peaks between 80 and 120 ms after stimulus onset (Näätänen and Picton, 1987). Neurophysiological evidence suggests that the N2a/MMN reflects matching of the incoming stimulus to its internally stored representation (or template) occurring temporally before stimulus categorization (Patel and Azzam, 2005). It is therefore thought to be a neurophysiological correlate of pre-attentive sensory stimulus discrimination (Näätänen and Alho, 1995) and automatic auditory change detection (Escera et al., 1998), even during passive auditory stimulus presentation (Näätänen et al., 2007). In response to pure tones, neural generators of the N2a/MMN can be found in multiple cortical areas, including auditory sensory cortices such as A1 and A2 (BAs 41/42), the dorsolateral prefrontal cortex (DLPFC; BAs 9/46), ACC (BAs 24/32/33) and the insular cortex (BA 13). In particular, the AAC is a cortical structure involved in self-related processing (Qin and Northoff, 2011), error processing (Bush et al., 2000) and attention modulation (Weissman et al., 2005), whereas the insula plays an essential role during allocation of auditory attention and detection of novel and salient auditory stimuli (Bamiou et al., 2003). More specifically, both regions are known to be part of the ‘salience network’ (SN; Seeley et al., 2007; Uddin, 2014), which akin to the CMS is involved in the detection of stimulus salience and thereby being responsible for an automatic, adaptive and suitable (behavioral) response (Menon and Uddin, 2010). However, neurophysiological evidence that the aforementioned cortical structures and/or the SN are activated during the perception of self-related movement stimuli such as finger snapping sounds is still lacking.

Like the N2a/MMN, the P3 is elicited only in response to unexpected or salient stimuli (Squires et al., 1975). For acoustic stimuli such as pure tones, P3 amplitudes are most pronounced between 300 and 450 ms post-stimulus at central-parietal and parietal electrode sites (Polich and Kok, 1995). Generally, the elicitation of the P3 depends on whether the deviant stimulus is sufficiently salient from the standard stimulus to allocate and direct attention (Picton, 1992). As a result, the P3 is believed to reflect a voluntary switching mechanism of attention given adequate change (Escera et al., 1998, 2000) and target detection (Debener et al., 2005). Moreover, the P3 has been suggested to reflect a process based on memory updating which is guided by stimulus evaluation (Kok, 2001) and context updating (Donchin and Coles, 1988; Polich, 2007). Previous oddball studies using pure tones suggest that neural activation in the P3 window may partly originate in the parietal and temporal cortices (BAs 39/40) although generally the neural underpinnings of the P3 are still poorly understood (Polich, 2007) and may vary as a function of stimulus salience (e.g., see Downar et al., 2002).

As far as temporal processing is concerned, we expected that ERPs elicited during passive listening will discriminate previously recorded self- from other-related finger snapping sounds, possibly already at very early sensory processing stages. With regard to the previous literature, an important open question is, whether discrimination will already occur in the time windows where discrimination between deviant and frequent pure tone stimuli is possible (e.g., in the time window of the N1 and N2a/MMN) or whether discrimination between self- vs. other-related snapping sounds is possible only at later stages of information processing (e.g., in the time window of the P3).

In situations where fMRI is not accessible, electrotomographic methods such as sLORETA that model and localize brain electrical activity based on multi-channel EEG recordings have been very fruitful, even for localizing activity in medial cortical brain structures such as the ACC (e.g., see Pizzagalli et al., 2001), which are thought to play an imminent role in self-related processing (for a review, see Northoff and Bermpohl, 2004; Northoff et al., 2006). Methodologically, sLORETA has been developed to make assumptions about the location of neural generators underlying brain electrical activity by estimating the maximally smoothed linear inverse solution (Pascual-Marqui et al., 1994; Pascual-Marqui, 1999, 2002). The validation of the sLORETA algorithm has been confirmed by various studies including studies with combined fMRI-EEG (e.g., see Vitacco et al., 2002). More specifically, an elegant study by Mulert et al. (2004) employed a ‘classical’ oddball paradigm with pure tones to compare brain activations measured by fMRI with those obtained using sLORETA. Results show that sLORETA successfully estimates sources of the underlying neural correlates of unimodal auditory stimuli with minimal localization error (which is due to the low-resolution of calculated sLORETA images). Accordingly, in the present study, sLORETA was used to estimate the neural sources underlying implicit discrimination of self- vs. other-related finger snapping sounds and to compare the activation patterns produced by these stimuli with those obtained for pure tones in the oddball paradigm. In particular, extending this previous research will help to explore whether brain regions belonging to the CMS are also involved in implicit processing of self-related movement sounds when the movement itself is not executed and whether listening to these sounds leads to stronger activity increases in the IFG and sensorimotor regions of the mirror neuron systems for sounds belonging to one’s own vs. another’s snapping movements.

## Materials and Methods

### Participants

Twelve university students (seven females, five males) between 19 and 26 years old (*M* = 21.3 years, *SD* = 2.15) of the German Sports University Cologne participated in the present study. All participants were in good health and reported no psychological or hearing disorders. Nine participants were right-handed according to the Edinburgh Handiness Inventory (Oldfield, 1971). All participants were naïve concerning the hypotheses of the experiment and had no previous experience with participation in a similar experiment. The experiment complied with the Declaration of Helsinki and was approved by the local ethics committee. Furthermore, all participants gave written informed consent before the start of the experiment and received monetary compensation (12 Euros) for their participation. The experiment (including EEG recordings) had been conducted at the Institute of Psychology of the German Sport University Cologne, Germany.

### General Procedure

Individual finger snapping sounds were recorded for each participant. They were told that the recordings would be used to build-up a standardized database containing natural movement sounds to be used in future studies (see also Graux et al., 2013, 2015 for a similar cover story). The recorded sound files were edited by the experimenter while participants answered questions about their handedness and health. Subsequently, participants were prepared for the EEG recording session. Before the start of the EEG session, participants had been seated in a comfortable chair and were informed about the general procedures of EEG recordings and the experiment. The experiment contained in total three different blocks. It always started with the passive pure tone oddball paradigm (block 1), which was followed by two blocks of the passive ‘Self-Other’ oddball paradigm (blocks 2 and 3). Block order of blocks 2 and 3 was counter-balanced across participants. The time interval between recording of each participant’s individual finger snapping sound and the start of block 2 was about 45 min. After the experiment, participants were invited to answer open as well as closed questions regarding the experimental set-up including their ability to recognize their own previously recorded finger snapping sounds. Finally, participants were debriefed regarding the purpose of the experiment.

### Stimulus Generation

Individual finger snapping sounds were recorded using a stationary Zoom H1 sound recorder (Zoom, Tokyo, Japan) mounted on a small MAGNESIT COPTER CB2.7 tripod (Cullmann Germany GmbH, Langenzenn, Germany). Participants sat centric in front of the sound recorder, with a distance of approximately 30 cm. Each participant was instructed to perform 10 finger snaps in total using his/her dominant hand with an interval of approximately 1 s to avoid overlapping sound recordings. The sound of a finger snap was created by rapidly forcing out air between two fingers (usually thumb and middle finger) with a characteristic audible snap. Intra- and inter-individual cross-correlations revealed significant correlations across all 10 individual finger snapping sounds (Spearman rho all *p* < 0.01), whereas inter-individual finger snapping sounds were – except from few exceptions – uncorrelated. In addition, individual finger snapping sounds showed high internal consistency (Kronbach’s alpha = 0.92). Nevertheless, to avoid physical differences between individual finger snapping sounds that could yield different effects in the averaged ERP waveforms, only one of the 10 finger snapping sounds per participant was chosen as actual experimental stimulus. This individual snapping sound was chosen from snaps 4–7, to exclude possible variability in finger snapping (finger snaps 1–3 or 8–10) due to fatigue or familiarization. Additionally, the individually chosen sound recordings yielded the best auditory quality. Raw sound files (PCM, stereo, 32-bit float, and 44.100 Hz) were edited oﬄine using the Audacity 1.2.6 package^2^. Editing of sound files included cutting (final experimental stimulus duration = 300 ms), applying a fade-in/out (10 ms), and normalizing (equal peak amplitude = 0 dB). Used sound stimuli are available upon request.

### Tone Oddball Paradigm (Block 1)

The standard tone oddball paradigm (as described in Williams et al., 2005) consisted of one block with two stimuli, a low (frequency: 500 Hz) pure tone as “standard” and a high (frequency: 1000 Hz) pure tone as “deviant,” with the following stimulus properties: pulse-code modulation (PCM), stereo, 32-bit float, and 44.100 Hz, stimulus duration = 50 ms, fade-in/out = 5 ms and normalized equal peak amplitude = 0 dB. Hence, both pure tones differed only in the frequency domain. The paradigm consisted of total of 400 trials (325 standard trials and 75 deviant trials, respectively) with an unjittered inter-stimulus-interval (ISI) of 950 ms. According to Williams et al. (2005), the application of an unjittered ISI has been found to elicit a reliable and robust N1 component in response to deviant and standard pure tones as well as a P3 component in response to deviant pure tones only. The presentation of the stimuli was controlled by the Inquisit 4.0 software package (Millisecond Software, Seattle, WA, USA). The experimental script was downloaded from the official Inquisit website^3^.

### “Self-Other” Oddball Paradigm (Blocks 2 and 3)

For every participant, his/her own finger snapping sound served as the self-related sound and the finger snapping sound of another participant was chosen as the non-self-related sound. More specifically, the “Self-Other” oddball paradigm consisted of two blocks with “Self” as standard and “Other” as deviant stimuli and vice versa. Each of the two blocks consisted in total of 400 trials (325 standard trials and 75 deviant) with an ISI of 700 ms. Block 1 (tone oddball paradigm) differed from blocks 2 and 3 only on the differences in ISI and the stimulus material used (pure tones vs. previously recorded finger snapping sounds). The whole paradigm with all three experimental blocks lasted for about 25–30 min (including short breaks).

For experimental purposes, participants had been grouped into matching gender pairs. Due to the use of such an experimental design, averaged ERPs to physically identical stimuli could be compared in self-related vs. other-related sound conditions. On the physical level, all finger snapping sound pairs were matched on fundamental frequency (f_0_). The mean frequency difference in f_0_ between self- (*M* = 2532.75 Hz, *SD* = 481.99) and other-related (*M* = 2520.42 Hz, *SD* = 378.67) finger snapping sounds did not differ significantly from each other, *t*(11) = -0.07, *p* = 0.944. Hence, self-/other-relatedness were the only stimulus parameters that could account for differences in all dependent ERP measurements during blocks 2 and 3.

### Stimulus Presentation

Auditory stimuli were presented via Shure SHR440 on-ear headphones (Shure, Niles, IL, USA). A sound level of about 75 dB/SPL was set for every participant (as measured in advance with a sound meter). As passive oddball paradigms address implicit stimulus processing, participants were told just to listen passively to the presented auditory stimuli (including pure tones and the previously recorded self-/other-related finger snapping sounds, respectively). Accordingly, no behavioral response was required. Furthermore, participants were instructed to fixate their view on a fixation cross presented on the video screen to avoid massive eye blinks while listening to the auditory stimuli.

### EEG Recordings

Continuous EEG data with a sampling frequency of 2.048 Hz were recorded from 64 Ag/AgCl sintered electrode sites arranged according to the international 10/10-system (Jurcak et al., 2007) using a Waveguard EEG cap (Advanced Neuro Technology B.V., Enschede, The Netherlands). The following electrode sites were used: FP1, FPz, FP2, AF7, AF3, AF4, AF8, F7, F5, F3, F1, Fz, F2, F4, F6, F8, FT7, FC5, FC3, FC1, FCz, FC2, FC4, FC6, FT8, T7, C5, C3, C1, Cz, C2, C4, C6, T8, TP7, CP5, CP3, CP1, CPz, CP2, CP4, CP6, TP8, P7, P5, P3, P1, Pz, P2, P4, P6, P8, PO7, PO5, PO3, POz, PO4, PO6, PO8, O1, Oz, O2, M1, and M2. During all EEG recordings, no oﬄine or stationary filters were used. All EEG channels were referenced to the common average of all scalp electrodes. The forehead electrode AFz served as ground electrode. Horizontal and vertical electrooculography data (HEOG and VEOG) were recorded using Blue Sensor N disk electrodes (Ambu, Ballerup, Denmark) placed at the outer canthi of the right and left eye, as well as below the left eye, respectively. All electrode impedances were kept below 10 kOhm.

### Pre-processing of Electrophysiological Data

Raw EEG data were edited oﬄine with the ASALAB (Version: 4.7.8) software package (Zanow and Knösche, 2004) before the data were further analyzed in MATLAB (Version: R2013a, 8.1.0.604; The MathWorks, Inc., Natick, MA, USA) in combination with the EEGLAB software toolbox^4^ (Version: 13.4.4b; Delorme and Makeig, 2004). During the EEG data analysis in ASALAB, a band-stop (notch) filter of 50 Hz (24 dB/oct) and a band-pass filter between 0.5 and 20 Hz (24 dB/oct) were used. Eye blinks and saccade-related artifacts were corrected with an artifact correction feature based on principal component analysis (PCA) as introduced by Ille et al. (2002). Further data analysis of artifact-free EEG data in EEGLAB involved down-sampling to 512 Hz, re-referencing to linked mastoids/linked ears (M1 and M2), segmentation into epochs for each condition [“Self” as standard and “Other “as deviant and vice versa, resulting in four different averaged conditions; hereafter, “SSt” ( = “Self Standard”), “OSt” ( = “Other Standard”), “SDe” ( = “Self Deviant”), and “ODe” ( = “Other Deviant”)] from 100 ms before and 700 ms after stimulus onset. All extracted epochs were baseline corrected 100 ms before stimulus onset.

### ERP Analysis and Statistics

Event-related potentials were analyzed in the P1, N1, N2, and P3 time windows. Statistically, significant differences in these time windows were identified by comparing averaged ERPs of the different experimental conditions with the built-in EEGLAB function “statcond” (Delorme, 2006) assuming the null hypotheses that there were no differences between experimental conditions.

For the statistical analysis of the pure tone oddball paradigm (block 1), averaged ERPs to rare and frequent stimuli (“Deviants” and “Standards,” respectively) were submitted to a non-parametric paired *t*-test with 5.000 permutations at all time points between 0 (stimulus onset) and 700 ms after stimulus onset with 62 electrode sites included.

To detect reliable statistical differences between averaged ERPs during the passive “Self-Other” oddball paradigm (blocks 2 and 3, respectively), averaged ERPs obtained from all four conditions (“SDe,” “SSt,” “ODe,” and ”OSt,” respectively) were submitted to a 1x4 repeated measures analysis of variance (ANOVA) with 5.000 permutations (including all time points between 0 and 700 ms post-stimulus and 62 electrode sites).

To further investigate statistical differences between the two experimental conditions presented during blocks 2 and 3 (“SDe” vs. “OSt” and “ODe” vs. “SSt,” respectively), averaged ERPs were repeatedly submitted to two non-parametric paired *t*-test with 5.000 permutations (including all time points between 0 and 700 ms post-stimulus and 62 electrode sites).

For all statistical analyzes, the false discovery rate (FDR; for an introduction, see Benjamini and Hochberg, 1995) was used to control for multiple comparisons, as implemented in the EEGLAB function “FDR.” The FDR-level was set to 5% (*q* = 0.05). The FDR procedure guarantees that the true FDR will be less than or equal to the nominal FDR level of 5% regardless of the dependency structure of the multiple tests (Benjamini and Yekutieli, 2001). Hence, the FDR procedure provides a much better spatial and temporal resolution by maintaining reasonable limits on the likelihood of false discoveries (i.e., it is suitable for a reasonable correction on a large number of comparisons) as compared to parametric *t*-test corrected using Bonferroni (for an introduction, see Lage-Castellanos et al., 2010).

To confirm the results obtained with EEGLAB, difference waves were calculated with the Maas Univariate ERP Toolbox ^5^ (Groppe et al., 2011a,b) to extract the MMN from the train of deviant and standard stimuli. The MMN was calculated and statistically compared for the contrast “SDe” – “OSt” and contrast “ODe” – “SSt,” respectively. To detect reliable differences in these specific time windows, difference waves were submitted to a repeated measures, two-tailed cluster-based permutation test based on the cluster mass statistic (Bullmore et al., 1999) using a family wise alpha level of 0.05. All time points in the time windows from 50–150 ms, 150–250 ms, and 250–450 ms, as well as all 62 scalp electrodes, were included in the statistical tests (i.e., 3.224, 3.224, and 7.998 total comparisons, respectively).

All repeated measures *t*-test were performed for each comparison using the original data and 2500 random within-participant permutations of the data. For each permutation, all *t*-scores corresponding to uncorrected *p*-values of 0.05 of less were formed into clusters with any neighboring such *t*-scores. Electrodes within approximately 5.44 cm of one another were considered spatial neighbors and adjacent time points were considered temporal neighbors. The sum of the *t*-scores in each cluster is the ”mass” of that cluster and the most extreme cluster mass in each of the 2.501 sets of tests was recorded and used to estimate the distribution of the null hypothesis (i.e., no difference between conditions^6^). The permutation cluster mass percentile ranking of each cluster from the observed data was used to derive its *p*-value. The *p*-value of the cluster was assigned to each member of the cluster and *t*-scores that had been not included in a cluster were given a *p*-value of 1.

This permutation test analysis was used instead of more conventional mean amplitude ANOVAs because it provides much better spatial and temporal resolution than conventional ANOVAs while maintaining weak control of the family wise alpha level (i.e., it corrects for a large number of comparisons). Moreover, the cluster mass statistic was chosen for this permutation test because it has been shown to have relatively good power for broadly distributed ERP effects like the P3 (Maris and Oostenveld, 2007; Groppe et al., 2011b). 2.500 permutations were used to estimate the distribution of the null hypothesis as it is over twice the number recommend by Manly (1997) for a family-wise alpha level of 0.05.

### EEG Source Localization

In a subsequent analysis, neural generators of the averaged ERPs were analyzed with sLORETA software (University Hospital of Psychiatry, Zürich, Switzerland)^7^ in time intervals showing significant differences between experimental conditions (based on the maximum number of statistically significant differences as revealed by the Mass Univariate ERP Toolbox).

sLORETA uses a distributed source localization algorithm to solve the inverse problem of brain electric activity (for a technical review, see Pascual-Marqui et al., 2002) regardless of the final number of neural generators (Pascual-Marqui, 1999, 2002). The sLORETA algorithm calculates the current density values (unit: amperes per square meter; A/m^2^) of 6.239 gray matter voxels belonging to the brain compartment with a spatial resolution of 5 mm × 5 mm × 5 mm each. The whole three-dimensional brain compartment comprises cortical gray matter and the hippocampus only and does not contain any deep brain structures such as the thalamus or the cerebellum. Anatomical regions are labeled according to (1) the probabilistic MNI-152 template made digitally available by the Brain Imaging Center of the Montreal Neurological Institute (MNI; Mazziotta et al., 2001) and (2) the Talairach Daemon (Lancaster et al., 2000) – a digitized version of the Co-Planar Stereotaxic Atlas of the Human Brain introduced by Talairach and Tournoux (1988).

To display differences for all discrete time windows between the two experimental conditions (e.g., “Deviant” > “Standard” for the tone oddball paradigm), statistical non-parametric mapping (SnPM) as introduced by Nichols and Holmes (2002) was used to compute the averaged intracerebral current density distribution at time intervals showing significant differences based on non-parametric voxel-by-voxel one-tailored paired samples *t*-test (with 5.000 permutations) on the three-dimensional sLORETA images. Statistical significance was assessed by defining critical thresholds (*t*_crit_) corrected for multiple comparisons (*p* < 0.01 and *p* < 0.05, respectively) for all tested voxels and time windows. The null hypotheses equaled the assumption that there were no differences among experimental conditions. Current density values at each voxel have been computed in the solution space as a linear and weighted sum of the scalp electric potentials. Activation of a given voxel was based on the smoothness assumption, meaning that neighboring voxels show a highly synchronous activity (Silva et al., 1991). Support comes from electrophysiological studies showing that neighboring neural populations show a highly correlated electrical activity (Silva et al., 1991; Haalman and Vaadia, 1997). As proposed by Friston (2002) activated voxels exceeding *t*_crit_ were considered as being regions of cortical activation. Finally, statistical analysis resulted in an averaged corresponding three-dimensional intracerebral current density distribution and obtained cortical regions were classified about their corresponding BA (Brodmann and Gary, 2006) and normalized coordinates (Talairach and MMI, respectively).

## Results

### Post-test Questions

Open questions after blocks 2 and 3 revealed that 10 out of 12 participants identified the presented sounds as finger snapping sounds. Furthermore, the same participants were sure that one of their previously recorded finger snapping sounds were used as experimental stimulus, although when later on explicitly asked to recognize their own snapping sound, only four participants correctly identified their previously recorded finger snapping sound.

### ERPs – Tone Oddball Paradigm

**FIGURE 1:**
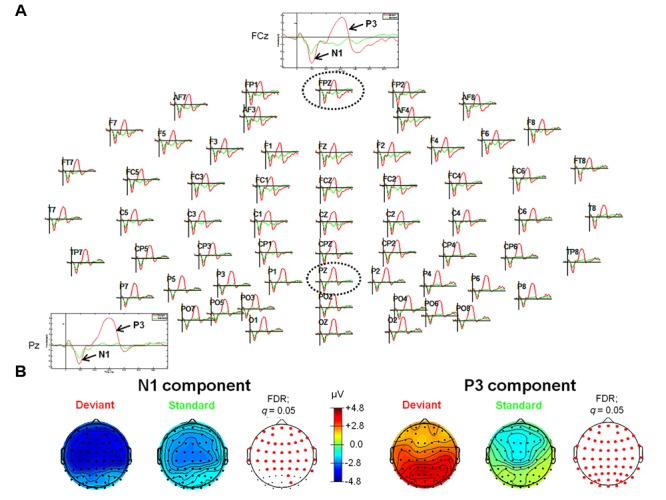
**Event-related potentials (ERPs; upper panel, **A**) and topographic plots (lower panel, **B**) for the N1 (lower panel, left) and P3 (lower panel, right) components in the experimental conditions of the tone oddball paradigm. (A)** The head plot shows ERP waveforms from 62 electrode sites. One anterior electrode site (FCz) as well as one posterior electrode site (Pz) are shown in detail (as indicated by the black dashed circles). ERP waveform plots reveal the N1 and the P3 components with significantly higher amplitudes in response to deviant stimuli (pure tones with a frequency of 1000 Hz) as compared to standard stimuli (pure tones with a frequency of 500 Hz). **(B)** Reddish colors indicate positive ERP values, whereas bluish colors indicate negative ERP values. In addition, transparent EEG montage arrays (lower panel **B**) show statistically significant electrode sites as indicated by red dots (after comparison for multiple comparisons with FDR). Obtained time windows have been averaged between 82–129 ms (N1 component) and 233–358 ms (P3 component), respectively. FDR, False discovery rate.

For the tone oddball paradigm, ERP waveforms of deviant and standard tones included the following ERP components: N1 (peak: at about 95 ms) and P3 (peak: at about 300 ms), respectively (see **Figure 1**). Statistical results obtained with EEGLAB revealed two different time windows during which ERPs showed significant differences between deviants and standard tones. The retrieved two time windows were found between 82 and 129 ms and between 233 and 358 ms post-stimulus (see **Figure 1** for an overview).

### Difference Wave – Tone Oddball Paradigm

Statistical results obtained with the Mass Univariate ERP Toolbox confirmed two different time windows during which the computed difference wave (“Deviant” minus “Standard”) showed significant differences. For the first time window (equivalent to the elicited N1 component), statistically significant differences were found between 72 and 138 ms post-stimulus. The maximum number of statistically significant differences was observed between 84 and 129 ms post-stimulus at 14 electrode sites, including FP2, F4, F8, FC2, FC6, C4, T8, AF4, F2, F6, FCz, FC4, C6, and FT8. This negative deflection seems to mimic an ‘early’ MMN (Luck, 2005) peaking at about 120 ms. To ensure the validity of this interpretation, all connected electrodes were re-referenced to a common average reference (CAR). According to the literature available, CAR or a nose reference are recommended as these montages are known to be the best reference sites to robustly determine the MMN (Koelsch, 2012). As expected, this procedure confirmed the characteristic polarity inversion of the extracted MMN at both mastoid electrodes sites (M1 and M2, respectively). Thus, the extracted MMN of the difference wave overlapped with the elicited N1 component observed in the averaged ERP waveforms (e.g., see Campbell et al., 2007). For the second time window, statistically significant differences were found between 203 and 363 ms after stimulus onset. The maximum number of statistically significant differences was observed between 242 and 344 ms after stimulus onset and was observed at all 62 electrode sites. This positive deflection peaking at about 295 ms was interpreted as P3 component (Luck, 2005).

### ERPs – “Self-Other” Oddball Paradigm

**FIGURE 2:**
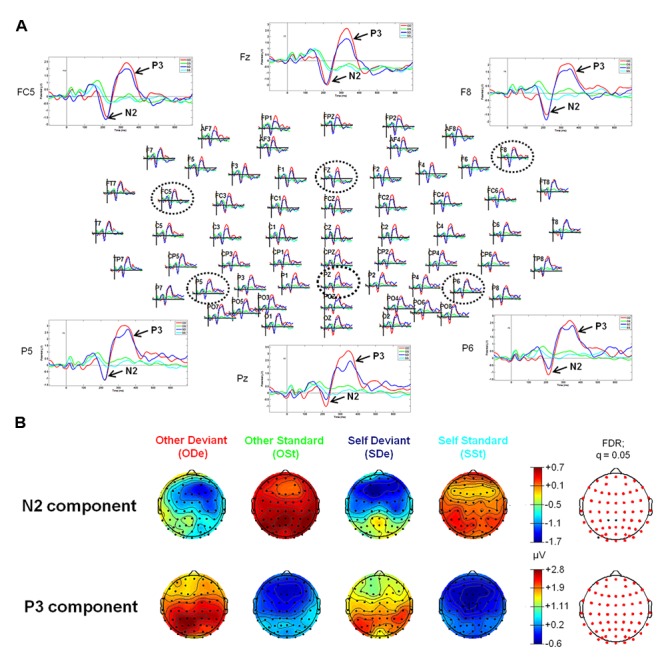
**Event-related potentials (upper panel, **A**) and topographic plots (lower panel, **B**) for the N2 and P3 components in all four experimental conditions of the “Self-Other” oddball paradigm. (A)** The head plot shows ERP waveforms from 62 electrode sites. Three anterior electrode sites (FC5, Fz, and F8) as well as three posterior electrodes (P5, Pz, and P6) are shown in detail (as indicated by black dashed circles). ERP waveform plots reveal the N2-P3 complex. Higher amplitudes of the N2 component can be observed at anterior sides, whereas the P3 shows higher amplitudes at posterior electrode sites (especially in condition “ODe”). **(B)** Reddish colors indicate positive ERP values, whereas bluish colors indicate negative ERP values. Moreover, transparent EEG montage arrays (lower panel **B**) show statistically significant electrode sites as indicated by red dots (after comparison for multiple comparisons with FDR). FDR, False discovery rate.

For the “Self-Other” oddball paradigm, visual inspection of ERP waveforms of both deviant stimuli, “SDe” and “ODe” revealed the following ERP components: P1, N2, P3 (the so-called “N2-P3 complex”) peaking at about 155, 215, and 330 ms, respectively. In contrast to the tone oddball paradigm, no clear N1 was observed in response to “SDe” as well as “ODe” deviant stimuli. This finding is in accordance with electrophysiological studies indicating that a reduction of the ISI (950 vs. 700 ms during the tone and “Self-Other” oddball paradigm, respectively) leads to a decrease in N1 amplitude (e.g., see Davis et al., 1966; Nelson and Lassman, 1968; Fitzgerald and Picton, 1981; Alcaini et al., 1994; Pereira et al., 2014). Furthermore, the amplitude of the auditory N1 is highly determined by stimulus parameters such as frequency and amplitude of the presented stimuli. As supported by several parametric studies, the amplitude and latency of the N1 decrease as stimulus frequency increases. In the extreme case, the N1 amplitude diminishes particularly at frequencies higher than 2000 Hz (e.g., see Antinoro et al., 1969; Wunderlich and Cone-Wesson, 2001). Given that the recorded finger snapping sounds in our study yield a mean fundamental frequency (f_0_) above 2000 Hz [see “Self-Other” Oddball Paradigm (Blocks 2 and 3)], it is highly plausible that the same is true for the finger snapping sounds used in the present study. Additionally, no clear N2 and P3 components were observed for both standard stimuli (“OSt” and “SSt,” respectively), see **Figure 2.**

Statistical results obtained with EEGLAB revealed two distinct time windows during which ERPs showed significant differences between all four experimental conditions (“ODe,” “SDe,” “OSt,” and “SSt,” respectively). No significant effects were found in earlier time windows (e.g., P1 or N1). For the comparison between the experimental condition “SDe” and “OSt,” statistical results revealed two distinct significantly different time windows, namely between 180 and 219 ms and between 279 and 385 ms post-stimulus. In contrast, the statistical test comparing “ODe” and “SSt” showed only one significant time window, namely between 260 and 395 ms after stimulus onset. No significant effects were found in earlier time windows (e.g., P1 or N1).

### Difference Waves – “Self-Other” Oddball Paradigm

**FIGURE 3:**
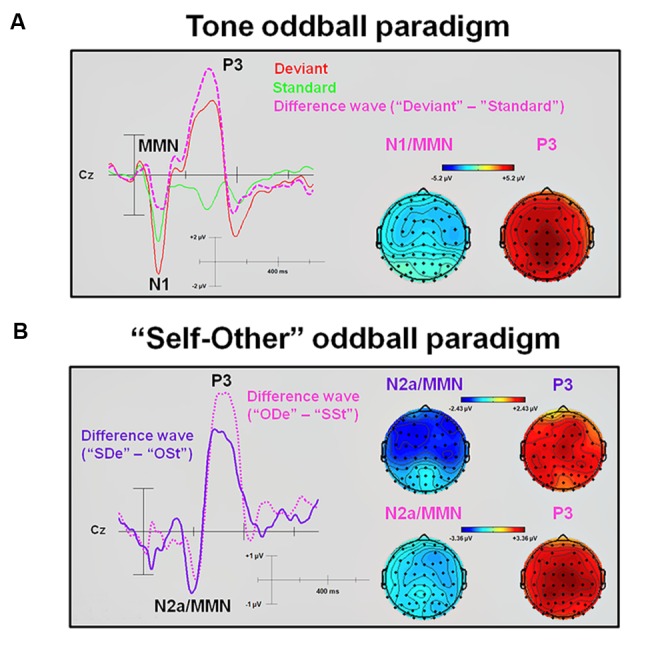
**Difference waveforms and corresponding topographic plots obtained from subtracting standards from deviants in the tone and the “Self-Other” oddball paradigm. (A)** An averaged ERP waveform and an extracted difference wave (“Deviant” minus “Standard”) at electrode site Cz. In addition, topographic plots of the N1/MMN and P3 peaks are shown. **(B)** Extracted difference waves (“SDe” minus “OSt” and “ODe” minus “SSt,” respectively) at electrode site Cz. In addition, topographic plots of the N2a/MMN and P3 peaks are shown. MMN, Mismatch negativity.

Statistical results obtained with the Mass Univariate ERP Toolbox confirmed two different time windows during which the extracted “SDe” minus “OSt” difference wave showed significant differences. For the first time window, statistically significant differences were found between 166 and 232 ms post-stimulus (see **Figure 3**). The maximum number of statistically significant differences was observed between 180 and 219 ms post-stimulus at 13 electrode sites, including F7, F3, FZ, F8, FC5, FC1, C4, AF7, AF3, F5, F1, C5, and FT7. This negative deflection was interpreted as N2a or MMN (Luck, 2005), with a peak at about 215 ms. Similar to the tone oddball paradigm, re-referencing to CAR confirmed the characteristic polarity inversion of the extracted MMN at the left and right mastoid electrodes sites (M1 and M2, respectively). For the second time window, statistically significant differences were found between 260 and 404 ms after stimulus onset. The maximum number of statistically significant differences was observed between 295 and 367 ms after stimulus onset and was observed at 51 electrode sites (F3, FZ, F4, F8, FC1, FC2, FC6, C3, CZ, C4, T8, CP5, CP1, CP2, CP6, P7, P3, P4, P8, O1, AF7, AF3, AF4, AF8, F5, F1, F2, F6, FC3, FCZ, FC4, C5, C1, C2, C6, CP3, CPZ, CP4, P5, P1, P2, P6, PO5, PO3, PO4, PO6, FT8, TP7, TP8, PO7, and PO8). This positive deflection corresponds to the P3 component (Luck, 2005), with a peak at about 355 ms. In contrast, the statistical test comparing the extracted “ODe” minus “SSt” difference wave showed only one significant time window, namely between 256 and 402 ms after stimulus onset. The maximum number of statistically significant differences was observed between 275 and 387 ms post-stimulus at all 62 electrode sites. Again, this positive deflection (peaking at about 335 ms) corresponded to the P3 component (Luck, 2005), see **Figure 3**. Hence, the statistical test in the corresponding averaged time window of the N2a/MMN component indicated no significant difference when previously recorded other-related finger snapping sounds were presented as the deviant stimuli and the participants’ previously recorded self-related finger snapping sounds were presented as standard stimuli. No significant differences were found between deviant (“ODe” and “SDe”) and standard (“OSt” and “SSt”) conditions, respectively. For a complete overview of all retrieved statistically significant results including all calculated contrasts, see **Tables 1A**,**B**.

**Table 1:** **Overview of all retrieved statistically significant results (non-parametric ANOVA or *t*-test with 5.000 permutations, FDR-corrected, *q* = 0.05) in the time windows corresponding to the N1, P1, N2a/MMN, and P3 component, respectively**.

				Component			
Experimental conditions				P1	N1	N2a/MMN	P3
**ERP contrasts (EEGLAB)**			
Self deviant (“SDe”)	Other standard (“OSt”)	Other deviant (“ODe”)	Self standard (“SSt”)	n.s.	n.s.	181–223 ms	267–391 ms
Self deviant (“SDe”)	Other standard (“OSt”)			n.s.	n.s.	179–220 ms	279–385 ms
Other deviant (“ODe”)	Self standard (“SSt”)			n.s.	n.s.	n.s.	260–400 ms
Self deviant (“SDe”)	Self standard (“SSt”)			n.s.	n.s.	193–221 ms	275–375 ms
Other deviant (“ODe”)	Other standard (“OSt”)			n.s.	n.s.	189–221 ms	274–386 ms
Self deviant (“SDe”)	Other deviant (“ODe”)			n.s.	n.s.	n.s.	n.s.
Self standard (“SSt”)	Other standard (“OSt”)			n.s.	n.s.	n.s.	n.s.

*ANOVA, analysis of variance; n.s., not significant; ms, milliseconds.*

**Table 1B:** Overview of the statistically significant results for the extracted difference waves in the time windows corresponding to the N2a/MMN and P3 component, respectively.

		Component	
Experimental conditions		N2a/MMN	P3
**Difference waves (Mass Univariate ERP Toolbox)**			
Self deviant (“SDe”)	Other Standard (“OSt”)	180–219 ms	260–404 ms
Other deviant (“ODe”)	Self Standard (“SSt”)	n.s.	256–402 ms

*ANOVA, analysis of variance; n.s., not significant; ms, milliseconds.*

### sLORETA Source Localization – Tone Oddball Paradigm

**FIGURE 4:**
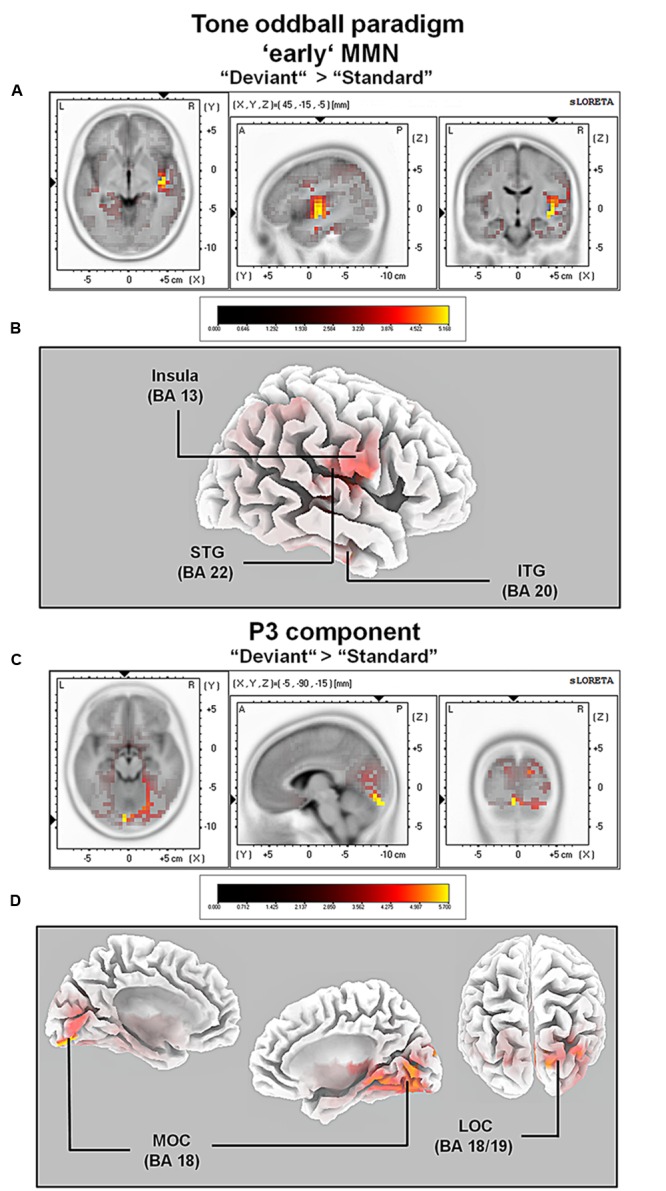
**Results of the standardized low-resolution brain electrotomography (sLORETA) source localization analysis (contrast: “Deviant” > Standard”) in the averaged time window of the ‘early’ MMN and P3 components (84–129 and 242–344 ms, respectively).** Images have been obtained after statistical non-parametric mapping (SnPM) and co-registration to the stereotaxic Talairach space based on the Co-Planar Stereotaxic Atlas of the Human Brain (Talairach and Tournoux, 1988) and the probabilistic MNI-152 template (Mazziotta et al., 2001). Activated voxels are indicated by yellowish and reddish colors [after correction for multiple comparisons (*p* < 0.01 and *p* < 0.05, respectively)]. **(A)** The peak of highest cortical activity has been found in parts of the superior temporal gyrus (STG; BA 22). **(B)** A shifted lateral view of the right hemisphere, showing cortical activations on the three-dimensionally rendered Colin27 template (Holmes et al., 1998). **(C)** The peak of highest cortical activity was found in the left medial occipital cortex (MOC; BA 19). **(D)** Two medial views on the left as well as the right hemisphere with cortical activations in the medial occipital cortices bilaterally and additionally in the right lateral occipital cortex (LOC; BAs 18 and 19), rendered on the Colin27 template (Holmes et al., 1998). L, left; R, right; A, anterior; P, posterior; MNI, Montreal Neurological Institute; X, Y, Z, corresponding MNI coordinates; BA, Brodmann area; MOC, medial occipital cortex; LOC, lateral occipital cortex; PCC, posterior cingulate cortex; ITG, inferior temporal gyrus.

For the averaged time window between 84 and 129 ms (corresponding to the ‘early’ MMN), a significantly higher cortical activation for deviant in contrast to standard pure tones was found in the following cortical areas: the right superior temporal gyrus (STG; BA 22), the right insula (BA 13), the right sub-gyral temporal lobe (BAs 20/21), the right inferior temporal gyrus (ITG; BA 20), the right pre- and post-central gyri (BAs 4/6/43), the transverse temporal gyrus (BAs 41/42) and the left fusiform gyrus (FFG; BAs 20/36), see **Figure 4**.

For the averaged time window between 242 and 344 ms (corresponding to the P3 component), a significantly higher cortical activation for deviant in contrast to standard pure tones was found in the following cortical areas: the bilateral lingual gyrus (BA 18), the right cuneus (BAs 17/18/19/23/30), the right fusiform gyrus (BAs 18/19/37), the right parahippocampal gyrus (BAs 19/27/30/36), the right posterior cingulate cortex (PCC; BA 30), the right middle temporal gyrus (BA 19) and the right superior occipital gyrus (BA 19) as shown in **Figure 4**. For a complete overview of all retrieved statistically significant results including all anatomical regions and activated voxels, see **Tables 2** and **3**.

**Table 2:** Standardized low-resolution brain electromagnetic tomography (sLORETA) results of maximal brain electrical activity for “Deviant” vs. “Standard” (500 vs. 1000 Hz pure tones) in the ‘early MMN’ time window.

				Coordinates (X, Y, Z)						*t*-value		
Structure	BA	Hemisphere	Lobe	Talairach			MNI			Max.	Min.	No. of activated voxels
**Brain region**												
Superior temporal gyrus	**22**	R	Temporal	45	-15	-3	45	-15	-5	5.69^∗∗^	4.51^∗∗^	16
Insula	**13**	R	Sub-lobar	45	-15	1	45	-15	0	5.67^∗∗^	4.49^∗∗^	22
Sub-gyral	20, **21**	R	Temporal	45	-10	-8	45	-10	-10	5.48^∗∗^	4.47^∗∗^	5
Inferior temporal gyrus	**20**	R	Temporal	50	-11	-25	50	-10	-30	5.28^∗∗^	-	1
Precentral gyrus	3, **4**, 6	R	Frontal	54	-4	14	55	-5	15	4.91^∗∗^	4.44^∗∗^	18
Post-central gyrus	**43**	R	Parietal	50	-14	15	50	-15	15	4.85^∗∗^	4.44^∗∗^	3
Transverse temporal gyrus	**41**, 42	R	Temporal	50	-19	10	50	-20	10	4.80^∗∗^	4.52^∗∗^	3
Fusiform gyrus	20, **36**	L	Temporal	-45	-40	-23	-45	-40	-30	4.56^∗∗^	4.44^∗∗^	5

*Talairach/MNI coordinates and *t*-values correspond to the peak activity in each brain region. Bold numbers indicate maximal brain electrical activity in the corresponding BA.*

*^∗∗^*p* < 0.01; L, left; R, right; N, number; min., minimum; max., maximum; BA, Brodmann area; MNI, Montreal Neurological Institute.*

**Table 3:** Standardized low-resolution brain electromagnetic tomography results of maximal brain electrical activity for “Deviant” vs. “Standard” (500 vs. 1000 Hz pure tones) in the averaged P3 time window.

				Coordinates (X, Y, Z)						*t*-value		
Structure	BA	Hemisphere	Lobe	Talairach			MNI			Max.	Min.	No. of activated voxels
**Brain region**												
Lingual gyrus	**18**	R/L	Occipital	-5	-88	-8	-5	-90	-15	5.16^∗∗^	3.97^∗^	48
Cuneus	17, 18, 19, **23**, 30	R	Occipital	10	-72	13	10	-75	10	4.87^∗^	3.93^∗^	14
Fusiform gyrus	18, **19**, 37	R	Occipital	25	-74	-13	25	-75	-20	4.58^∗^	3.96^∗^	16
Parahippocampal gyrus	**19**, 27, 30, 36	R	Limbic	20	-54	-6	20	-55	-10	4.56^∗^	3.91^∗^	21
Posterior cingulate	**30**	R	Limbic	10	-67	13	10	-70	10	4.53^∗^	4.52^∗^	2
^∗^	**19**	R	Occipital	15	-59	-5	15	-60	-10	4.48^∗^	-	1
Middle temporal gyrus	**19**	R	Temporal	40	-81	23	40	-85	20	4.47^∗^	4.21^∗^	2
Superior occipital gyrus	**19**	R	Occipital	35	-76	27	35	-80	25	4.13^∗^	3.97^∗^	3

*Talairach/MNI coordinates and *t*-values correspond to the peak activity in each brain region. Bold numbers indicate maximal brain electrical activity in the corresponding BA.*

*^∗∗^*p* < 0.01, ^∗^*p* < 0.05; L, left; R, right; N, number; min., minimum; max., maximum; BA, Brodmann area; MNI, Montreal Neurological Institute.*

### sLORETA Source Localization – “Self-Other” Oddball Paradigm

**FIGURE 5:**
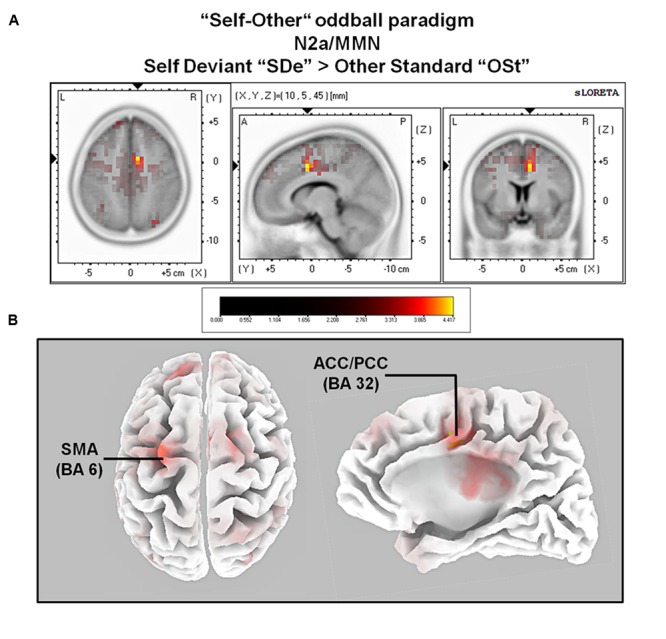
**Results of the sLORETA source localization analysis (contrast: “SDe” > “OSt”) in the averaged time window of the N2a/MMN component (180–219 ms).** Images have been obtained after SnPM and co-registration to the stereotaxic Talairach space based on the Co-Planar Stereotaxic Atlas of the Human Brain (Talairach and Tournoux, 1988) and the probabilistic MNI-152 template (Mazziotta et al., 2001). Activated voxels are indicated by yellowish and reddish colors [after correction for multiple comparisons (*p* < 0.01 and *p* < 0.05, respectively)]. **(A)** The peak of highest cortical activity was found in parts of the right ACC/PCC (BA 32) on the right medial surface of the brain. **(B)** A top view on the brain (left panel) shows highest cortical activity in the left supplementary motor area (SMA; BA 6). A lateral view on the right medial surface of the brain (right panel) shows cortical activations in the right ACC/PPC (BA 32), rendered on the Colin27 template (Holmes et al., 1998). L, left; R, right; A, anterior; P, posterior; MNI, Montreal Neurological Institute; X, Y, Z, corresponding MNI coordinates; BA, Brodmann area; SMA, supplementary motor area; ACC, anterior cingulate cortex; PCC, posterior cingulate cortex.

The comparison between experimental conditions “SDe” and “OSt,” in the averaged time window between 179 and 221 ms (corresponding to the N2a/MMN) revealed statistically higher cortical activations in the bilateral anterior cingulate gyri (BAs 23/24/31/32), the bilateral superior frontal gyri (BAs 6/8/9), the left superior temporal gyrus (BA 39), the bilateral middle frontal gyri (BA 6), the left posterior cingulate cortex (PPC; BA 23), the left superior temporal gyrus (BAs 22/39), the right medial frontal gyrus (BAs 6/32), the right superior parietal lobule (BA 7), the bilateral sub-gyral area (BA 6), the left inferior parietal lobule (BA 40), the left precuneus (BA 19/31), the precentral gyrus (BAs 6/9), the left insula (BA 13), the parahippocampal gyrus (BA 36) and the left fusiform gyrus (BA 37), see **Figure 5**. In the time window between 295 and 367 ms (corresponding to the P3 component) significantly higher cortical activations between experimental conditions “SDe” and “OSt” were observed in the right inferior parietal lobule (BA 40), the right precentral gyrus (BA 6), the right sub-gyral area (BA 6), the right superior frontal gyrus (BA 6), the left superior temoral gyrus (BA 41), the right middle frontal gyrus (BA 6), the left cuneus (BAs 28/34), the left insula (BA 13) and the left parahippocampal gyrus (BA 34), see **Figure 5**.

**FIGURE 6:**
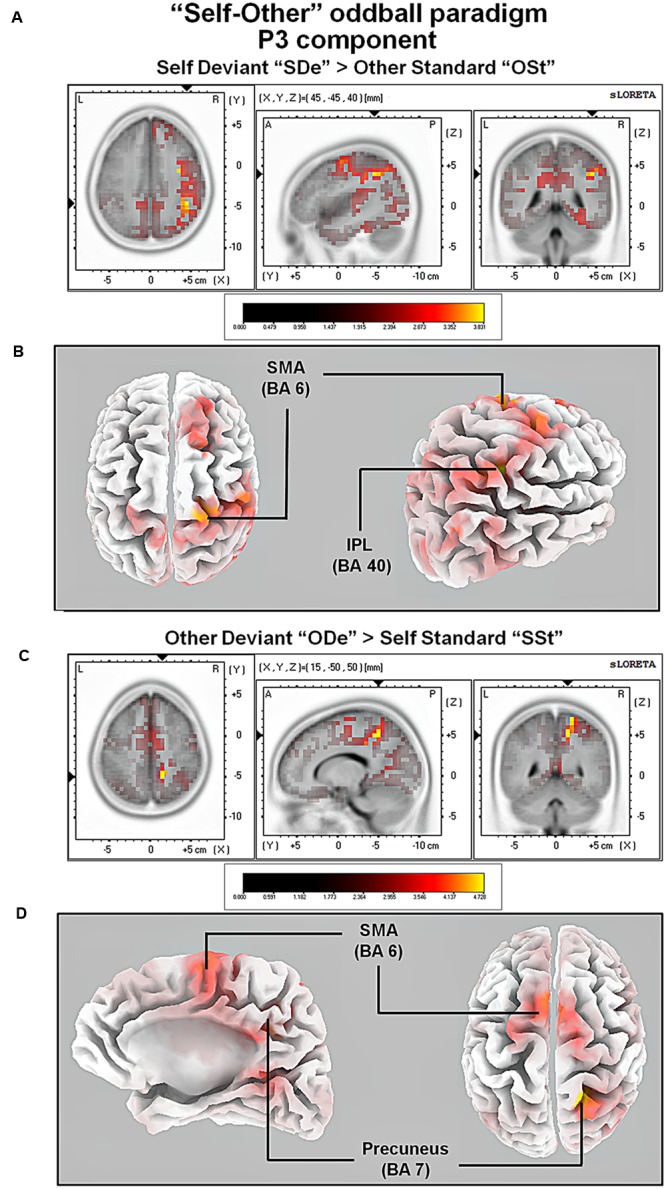
**Results of the sLORETA source localization analysis (contrasts: “SDe” > “OSt” and “ODe” > “SSt,” respectively) in the averaged time window of the P3 components (295–367 and 275–387 ms, respectively).** Images have been obtained after SnPM and co-registration to the stereotaxic Talairach space based on the Co-Planar Stereotaxic Atlas of the Human Brain (Talairach and Tournoux, 1988) and the probabilistic MNI-152 template (Mazziotta et al., 2001). Activated voxels are indicated by yellowish and reddish colors [after correction for multiple comparisons (*p* < 0.01 and *p* < 0.05, respectively)]. **(A)** The peak of highest cortical activity was found in the right inferior parietal lobule (IPL; BA 40). **(B)** A lateral view on the right hemisphere (right panel) and a top view on the whole brain (left panel) show highest cortical activations in the right IPL (BA 40) and the left supplementary area (SMA; BA 6) rendered on the Colin27 template (Holmes et al., 1998). **(C)** The peak of highest cortical activity was found in parts of the right precuneus (BA 7). **(D)** A top view on the whole brain (right panel) and additionally a medial view on right hemisphere (left panel) show cortical activations in the right precuneus (BA 7) and bilaterally in the supplementary motor area (SMA; BA 6) on the rendered Colin27 template (Holmes et al., 1998). L, left; R, right; A, anterior; P, posterior; MNI, Montreal Neurological Institute; X, Y, Z, corresponding MNI coordinates; BA, Brodmann area; TPJ, temporoparietal junction.

For the comparison between “ODe” and “SSt,” and the time window between 275 and 387 ms (corresponding to the P3 component) higher cortical activations were observed in the right precuneus (BAs 7/31), the right post-central gyrus (BAs 3/5/7), the right superior parietal lobule (SPL; BAs 5/7), the right paracentral lobule (BAs 5/31), the right sub-gyral area (BAs 7/40), the right cingulate gyrus (BAs 23/24/31), the bilateral superior and medial frontal gyri (BA 6) and the right posterior cingulate cortex (PPC; BAs 29/30), see **Figure 6**. For a complete overview of all retrieved statistically significant results including all anatomical regions and activated voxels, see **Tables 4–6**.

**Table 4:** Standardized low-resolution brain electromagnetic tomography results of maximal brain electrical activity for “SDe” vs. “OSt” in the averaged N2a/MMN time window.

				Coordinates (X, Y, Z)						*t*-value		
Structure	BA	Hemisphere	Lobe	Talairach			MNI			Max.	Min.	No. of activated voxels
Cingulate gyrus	23, 24, 31, **32**	R/L	Limbic	10	7	41	10	5	45	4.42^∗∗^	3.28^∗^	68
**Brain region**												
Superior frontal gyrus	**6**, 8, 9	R/L	Frontal	-25	-2	65	-25	-5	70	3.89^∗^	3.27^∗^	15
Middle temporal gyrus	39	L	Temporal	-50	-62	22	-50	-65	20	3.87^∗^	3.28^∗^	9
Middle frontal gyrus	**6**	R/L	Frontal	-25	-7	60	-25	-10	65	3.86^∗^	3.28^∗^	22
Posterior cingulate	**23**	L	Limbic	-5	-38	25	-5	-40	25	3.84^∗^	3.43^∗^	3
Superior temporal gyrus	22, **39**	L	Temporal	-45	-57	21	-45	-60	20	3.80^∗^	3.39^∗^	7
Medial frontal gyrus	**6**, 32	R	Frontal	10	7	50	10	5	55	3.68^∗^	3.29^∗^	13
Superior parietal lobule	7	R	Parietal	30	-71	45	30	-75	45	3.65^∗^	3.45^∗^	2
Sub-gyral	**6**	R/L	Frontal	-20	-7	56	-20	-10	60	3.59^∗^	3.32^∗^	5
Inferior parietal lobule	40	L	Parietal	-64	-42	25	-65	-45	25	3.50^∗^	3.29^∗^	7
Precuneus	19, **31**	L	Parietal	-20	-42	30	-20	-45	30	3.43^∗^	3.34^∗^	2
Precentral gyrus	**6**, 9	L	Frontal	-25	-12	56	-25	-15	60	3.38^∗^	3.27^∗^	5
Insula	**13**	L	Sub-lobar	-40	-43	21	-40	-45	20	3.36^∗^	3.36^∗^	2
Parahippocampal gyrus	**36**	L	Limbic	-20	-39	-6	-20	-40	-10	3.32^∗^	3.30^∗^	4
Fusiform gyrus	**37**	L	Temporal	-25	-40	-15	-25	-40	-20	3.29^∗^	-	1

*Talairach/MNI coordinates and *t*-values correspond to the peak activity in each brain region. Bold numbers indicate maximal brain electrical activity in the corresponding BA.*

*^∗∗^*p* < 0.01, ^∗^*p* < 0.05; L, left; R, right; N, number; min., minimum; max., maximum; BA, Brodmann area; MNI, Montreal Neurological Institute.*

**Table 5:** Standardized low-resolution brain electromagnetic tomography results of maximal brain electrical activity for “ODe” vs. “SSt” in the averaged P3 time window.

				Coordinates (X, Y, Z)						*t*-value		
Structure	BA	Hemisphere	Lobe	Talairach			MNI			Max.	Min.	No. of activated voxels
**Brain region**												
Inferior parietal lobule	**40**	R	Parietal	45	-42	39		-45	40	3.83^∗^	3.32^∗^	9
Precentral gyrus	**6**	R	Frontal	35	-3	37	35	-5	40	3.68^∗^	3.29^∗^	3
Sub-gyral	**6**	R	Frontal	35	-3	42	35	-5	45	3.62^∗^	-	1
Superior frontal gyrus	**6**	R	Frontal	20	-2	65	20	-5	70	3.61^∗^	-	3
Superior temporal gyrus	**41**	L	Temporal	-40	-34	6	-40	-35	5	3.53^∗^	-	1
Transverse temporal gyrus	**41**	L	Temporal	-35	-33	11	-35	-35	10	3.47^∗^	-	1
Middle frontal gyrus	6	R	Frontal	35	-8	42	35	-10	45	3.46^∗^	3.29^∗^	6
Uncus	**28**, 34	L	Limbic	-15	-1	-25	-15	0	-30	3.46^∗^	3.31^∗^	5
Insula	**13**	L	Sub-lobar	-30	-38	20	-30	-40	20	3.34^∗^	-	1
Parahippocampal gyrus	34	L	Limbic	-15	-1	-17	-15	0	-20	3.31^∗^	-	1

*Talairach/MNI coordinates and *t*-values correspond to the peak activity in each brain region. Bold numbers indicate maximal brain electrical activity in the corresponding BA.*

*^∗^*p* < 0.05; L, left; R, right; N, number; min., minimum; max., maximum; BA, Brodmann area; MNI, Montreal Neurological Institute.*

**Table 6:** Standardized low-resolution brain electromagnetic tomography results of maximal brain electrical activity for “SDe” vs. “OSt” in the averaged P3 time window.

				Coordinates (X, Y, Z)						*t*-value		
Structure	BA	Hemisphere	Lobe			Talairach	MNI			Max.	Min.	No. of activated voxels
**Brain region**									
Precuneus	**7**, 31	R	Parietal	15	-46	48	15	-50	50	4.73^∗∗^	3.37^∗^	8
Post-central gyrus	3, **5**, 7	R	Parietal	20	-45	67	20	-50	70	4.60^∗^	3.35^∗^	9
Superior parietal lobule	5, **7**	R	Parietal	20	-45	62	20	-50	65	4.53^∗∗^	3.37^∗^	10
Paracentral lobule	**5**, 31	R	Frontal/limbic	20	-41	53	20	-45	55	4.47^∗∗^	3.39^∗^	5
Sub-gyral	**7**, 40	R	Parietal	20	-46	53	20	-50	55	4.22^∗∗^	3.35^∗^	7
Cingulate gyrus	23, 24, **31**	R	Limbic	15	-32	38	15	-35	40	4.14^∗∗^	3.32^∗^	14
Superior frontal gyrus	**6**	R/L	Frontal	-5	13	64	-5	10	70	4.06^∗∗^	3.34^∗^	28
Medial frontal gyrus	**6**	R/L	Frontal	5	3	55	5	0	60	3.75^∗^	3.33^∗^	18
Posterior cingulate	29, **30**	R	Limbic	10	-53	16	10	-55	15	3.70^∗^	3.35^∗^	6

*Talairach/MNI coordinates and *t*-values correspond to the peak activity in each brain region. Bold numbers indicate maximal brain electrical activity in the corresponding BA.*

*^∗∗^*p* < 0.01, ^∗^*p* < 0.05; L, left; R, right; N, number; min., minimum; max., maximum; BA, Brodmann area; MNI, Montreal Neurological Institute.*

## Discussion

The present study examined the neural dynamics of implicit self-related processing of movement-related auditory information by using the sounds of participants’ own finger snapping sounds. Finger snapping sounds were recorded from each participant individually before the start of the EEG session. Participants first listened passively to pure tones (experimental block 1) and were then exposed to the previously recorded snapping sounds including their own vs. other finger snapping sounds (experimental blocks 2 and 3) in a passive listening oddball paradigm. Self vs. other-related finger snapping sounds were matched in f_0_ such that individually presented snapping sounds could differ only in self-/other-relatedness. Analyses of EEG data included (1) the identification of statistically significant differences in the averaged ERP waveforms between the different stimulus types (pure tones devoid of any personal or movement-related information vs. movement-related finger snapping sounds) and between self- vs. other-related finger snapping sounds and (2) the localization of significant differences with sLORETA.

### ERPs –Tone Oddball Paradigm – Time Course and Localization

Listening to tones elicited, as expected, two commonly known ERP components namely the N1 (including an early MMN as difference potential when deviants were contrasted against standards, see below) and the P3 component. Modulation of both ERP components (i.e., larger N1 and P3 amplitudes for deviant as compared to standard tones) was in line with several previously conducted ERP studies using a comparable passive auditory oddball paradigm with pure tones (e.g., for the N1 component see Zouridakis et al., 1998; Godey et al., 2001; for the P3 component, see Bennington and Polich, 1999; Patel and Azzam, 2005). Also in line with the previous literature, the P3 was elicited only by deviant pure tones (e.g., see Escera et al., 1998, 2000; Debener et al., 2005). Source localization with sLORETA revealed an increase in cortical activity in the right superior temporal gyrus (STG; BA 22) and the right insula (BA 13) in the time window of the N1 component corresponding to the ‘early’ MMN component (82–129 ms).

Experimental support for the obtained findings comes from an fMRI study conducted by Müller et al. (2003) using a comparable passive pure tone oddball paradigm. Results of this study confirm the involvement of cortical regions such as the right STG and insula during the processing of unimodal auditory deviant stimuli. Furthermore, as shown by Müller et al. (2003) during fMRI, the present EEG source imaging results confirm that insular activation is associated with processing and discrimination of simple auditory stimuli such as pure tones (for an introduction, see Bamiou et al., 2003) and thus not specific to a particular class of stimuli. In line with this, several neuroimaging studies have suggested that the insula is part of the ‘salience network’ (SN) and hence be activated during the detection of any type of novel or salient auditory stimuli (e.g., see Seeley et al., 2007; Uddin, 2014).

In addition to insula activation, deviant pure tones compared to standard tones elicited an increase in cortical activity in or adjunct to the right primary auditory cortex (A1; BA 42) including auditory association areas (A2; BA 42). These regions are situated in the right planum temporale (PT). Research findings suggest that the PT can be seen as a “computational hub” responsible for complex sound processing in the spectrotemporal domain (Griffiths and Warren, 2002), significantly involved in the modulation of attention (Woldorff et al., 1993). Additional cortical activations comprised the temporal lobe (e.g., STG; BA 22). These activation patterns are in close agreement with a previously conducted neuroimaging study combining fMRI with multi-channel EEG recordings during a passive auditory oddball paradigm (Liebenthal et al., 2003). Results of this study indicated cortical activations in the right superior temporal gyrus (STG; BA 22) and the right superior temporal plane (STP; BA 41 and 42), during deviance processing of pure tones in agreement with the present sLORETA source localization results.

Finally, activation of medial occipital and lateral occipital areas [e.g., parts of the right lateral occipital cortex (LOC); BAs 18/19] were observed in the time window of the P3 component Although occipital cortical regions are significantly involved in visual processing they are also involved in attention orientation (Grill-Spector et al., 1999, 2001) specifically in tasks requiring spatial attention (e.g., see Murray and Wojciulik, 2004). It is important to note that participants in the current study had been stimulated through the entire experiment with unimodal auditory information, except a centered black fixation cross on white background presented on a computer screen. That visual processing regions can be activated during the presentation of unimodal auditory stimuli has been demonstrated in several previous neuroimaging studies (e.g., see Bénar et al., 2007; Goldman et al., 2009). These studies combined simultaneous EEG and fMRI measurements and modeled the BOLD response obtained from fMRI, and the P3 obtained from the EEG during a comparable oddball paradigm with pure tones in the time window of the P3 component on a trial-to-trial basis. Results of these studies indicated that the P3 component was successfully localized in the aforementioned medial occipital and lateral occipital areas (Bénar et al., 2007; Goldman et al., 2009).

### ERPs – “Self-Other” Oddball Paradigm – Time Course

In contrast to pure tones, differences in the ERP waveforms in response to self- and other-related finger snapping sounds were first observed in the time window of the N2a/MMN (starting at about 180 ms post-stimulus) and thus temporarily after discrimination between deviant and frequent tones. As shown in **Figures 1**–**3**, in the self-other oddball tasks no clear N1 was observed in response to “SDe” or “ODe” deviant stimuli. Given that ISIs were shorter in the self-other oddball paradigm than the tone oddball paradigm and some studies suggest that N1 amplitudes are more pronounced for longer as opposed to shorter ISIs (Nelson and Lassman, 1968; Fitzgerald and Picton, 1981; Alcaini et al., 1994; Pereira et al., 2014), it is likely that this variation in ISI may have attenuated the N1 amplitude in the self-other oddball task. Nevertheless and remarkably, differential modulation of the N2a/MMN was only apparent when contrasting conditions “SDe” and “OSt” (contrast “SDe” > “OSt”), but did not emerge for the difference between conditions “ODe” and “SSt” (contrast “ODe” > “OSt”). This suggests that discrimination between self and other is attenuated or obscured when self-related stimuli are presented as standard stimuli lending support for implicit self-related processing of one’s own finger snapping sounds. Thus, self-related stimuli are akin to other salient self-related stimuli (e.g., own face, own name or own voice) processed in a facilitated manner even if listeners are not explicitly instructed to recognize the stimuli (e.g., see Graux et al., 2013, 2015). Of course, further EEG-ERP studies are needed to scrutinize preferential processing of self-related vs. other-related finger snapping sounds in larger population samples. This would also help validate the observation that in the present study other-related compared to self-related finger snapping sounds were not preferentially processed in early time windows when self-related finger snapping sounds were presented as standards and other-related finger snapping sounds as deviants in the oddball paradigm.

In the time window of the P3 component (starting at about 260 ms after stimulus onset), no difference in response to self- and other-related finger snapping sounds (contrasts “SDe” > “OSt” and “ODe” > “SSt,” respectively) was observed suggesting that the early elicitation of the N2a/MMN component, but not the later elicitation of the P3 component might be a fundamental neuro-physiological correlate for implicit discrimination of self- from other-related movement sounds. The N2a/MMN is believed to reflect continuous analysis of stimulus features, followed by updating and comparing information from an internally stored memory representation (for a review, see Patel and Azzam, 2005). Therefore, early sensory discrimination of one’s own finger snapping sounds in the time window of the N2a/MMN components might be associated with reduced memory update during later processing stages (P3 window), thereby attenuating the chance to find activity differences in the P3 time window for self- and other-related finger snapping sounds when these were presented as deviants.

### sLORETA – “Self-Other” Oddball Paradigm

When contrasting conditions “SDe” and “OSt” (contrast “SDe” > “OSt”) sLORETA source localization revealed increased cortical activity in the left lateral part of the primary motor cortex (M1; BA 6) and in parts of the right anterior cingulate/posterior parietal cortex (ACC/PPC; BAs 32/24) as early as in the N2a/MMN time window. Whereas motor regions are part of the mirror neuron system, the ACC/PCC are belonging to the CMSs (Northoff and Bermpohl, 2004). Hence, activation of motor mirror neurons and CMS in the time-window of the N2a/MMN component might reflect automatic (and thus implicit) processing of self-related movement-related auditory information, rather than conscious, reflective stimulus processing (Uddin et al., 2007; Esslen et al., 2008). Importantly, as outlined in more detail below, the results suggest that both systems (motor system as part of the mirror neuron system and CMS) are involved in implicit self-related processing and self-other discrimination.

In the P3 window, major neural sources of cortical activity for the contrast “SDe” > “OSt” included the right inferior parietal lobule [part of the right temporoparietal junction (TPJ; BA 39)] and the parts of the right supplementary area (SMA; BA 6). The involvement of the right TPJ is in agreement with a large body of evidence indicating that together with the aforementioned CMS, the rTPJ is one of the most important brain regions involved in self-other discrimination thereby facilitating self-related processing (Lou et al., 2004). In addition, it has been argued that besides being involved in unimodal visual or auditory stimulus processing, the right TPJ is important for integrating sensory information related to the self and self-other discrimination in general. In particular the right TPJ is assumed to establishes the phenomenological and cognitive aspects of the self, based on multimodal stimuli (Laureys, 2005).

We also contrasted the conditions “ODe” against “SSt” (contrast “ODe” > “SSt”) which revealed increased cortical activity in the supplementary motor area (SMA; BA 6) and parts of the right precuneus (BA 7). The involvement of the right precuneus during other-related processing is noteworthy, as this brain region belonging to the CMS has been repeatedly associated with self-related processing (e.g., Northoff and Bermpohl, 2004). Controversially, a recent meta-analysis by Qin and Northoff (2011) confirms not only an essential role of the right precuneus during self-related but also during other-related processing.

Whereas activation of CMS structures varied considerably across time windows and stimulus contrasts, activation in the SMA was observed in all three aforementioned contrasts. Especially for contrast “SDe” > “OSt,” increased cortical activity was found in an area in close proximity to the left (and thus contralateral) primary motor cortex (M1) already in the N2a/MMN time window. This finding is remarkable as 9 out of 12 participants of the current study were right-handed and also observed when left-handed participants were excluded from the analysis. An elegant fMRI study identified a proximate cortical region on the left cortical surface as being the location of the “motor hand area,” specifically activated during the execution of right hand and finger movements (Yousry et al., 1997). In addition, the current results are in line with previous research findings revealing similar cortical activation patterns in M1 contralateral to the participants’ dominant hand (Hauk et al., 2006). Based on the results of the current study, it can be concluded that increased cortical activity in different parts of primary and supplementary motor cortices (with cortical activations in the right and left hemisphere, respectively) has been triggered by the presentation of movement-related auditory information, regardless of whether this information was self- or other-related. Hence, in implicit processing designs as the present one M1 and SMA are not specifically involved in the processing of self-related movement information, but can be seen as an important “neural hub” facilitating the processing of this specific movement-related information when the movement itself is not executed during stimulus presentation. This assumption is also supported by the fact, that activations of M1 and SMA were absent in the tone oddball paradigm.

### Limitations and Future Outlook

A limitation of the present study might be the confounding factor of familiarity. Differences in familiarity between self- and other-related stimuli are of significant concern in research investigating self-related processing, especially with stimuli, such as the own name or face (e.g., see Graux et al., 2015). The own name or face are considered highly familiar stimuli recognized and identified even under adverse conditions. In contrast to the own name or face, a participant’s previously recorded self-related finger snapping sound can definitively be considered less familiar. Despite this and although differences in familiarity between self- and other-related snapping sounds had been reduced by our matching procedure (matching for sensory processing based on f_0_), familiarity effects cannot be fully excluded in the present study as the stimulus set was not explicitly tested for familiarity (for instance, by conduction a rating study assessing stimulus ratings of familiarity via self-report or by including an additional oddball paradigm with different types of familiar vs. unfamiliar sounds). Thus, whether such possible differences in stimulus familiarity actually contribute to neuro-physiological processing differences between self- and other-related finger snapping sounds, even during passive listening and thus implicit processing should be considered in future studies.

Yet, another issue to clarify is episodic memory. As mentioned in the present study, finger snapping sounds were recorded prior to the experiment. According to the manipulation check, only four participants correctly identified their previously recorded finger snapping sounds post-experimentally (see Post-test Questions). Thus, remembering as well as effects of episodic memory retrieval might have been low during stimulus presentation. Nevertheless, memory effects should be controlled for and investigated in future research as, so far, compared to studies on semantic memory little is known about memory for sensory information related to previously recorded self-related movement sounds.

Generally, the results of EEG source localization techniques should be interpreted with caution, although the retrieved cortical activation patterns of the present study are very plausible allowing us to integrate the results with those obtained from prior EEG and fMRI studies. Especially by combining sLORETA with ERP analysis allowed us to demonstrate when during stimulus processing and roughly where in the brain implicit self-recognition of auditory movement-related information takes place as this question has been largely unexplored even in prior research interested in the processing of self-related movement sounds (e.g., Aziz-Zadeh et al., 2004; Pizzamiglio et al., 2005; Hauk et al., 2006; Justen et al., 2014).

Of course, the current sLORETA source localization analysis is based on correlational statistics and hence results are only of correlational nature. The application of repetitive transcranial magnetic stimulation (rTMS) could offer an elegant and non-invasive way to selectively block or stimulate cortical activity in superficial brain regions (e.g., the right TPJ or motor areas) by applying fast trains of electromagnetic pulses (Walsh and Cowey, 2000). As such, rTMS studies would offer the potential to unravel causal relationships between cortical regions of interest (CMS and motor areas), involved in implicit processing of previously recorded self-related movement sounds.

## Conclusion

By investigating the temporal and the spatial processing dynamics of previously recorded self- and other-related movement sounds and by comparing the results with those obtained during listening to movement-unrelated deviant and frequent auditory stimuli in the same subjects, the present study determined the time-course of self-other discrimination and the implicit identification of previously recorded self-related finger snapping sounds. This revealed early discrimination between self- and other-related snapping sounds in the N2a/MMN time window and differential activation of brain structures as compared to pure tone processing. To our knowledge, it has never been shown before, how early the human brain distinguishes movement-related auditory information that has been generated by the self or the other but the movement itself is not executed during stimulus presentation. The present results suggest that one’s own finger snapping sounds can be distinguished from those belonging to another individual at an early auditory information processing stage, even if the movement is not executed at the time of testing. This suggests that preferential processing of the characteristic sensory features of self-related movement sounds occurs pre-reflectively and without participants being aware of it. Although, preferential processing of this kind has been described theoretically (e.g., see Legrand and Ruby, 2009) and empirically demonstrated in some studies using self-related stimuli, the present study is the first to confirm such a mechanism during implicit processing of sensory information belonging to self-related movements. In line with this, we could demonstrate that regions of the CMS and motor areas are involved in the processing of self-generated movement-related sounds and discussed their potential role in this type of processing.

## Author Contributions

CJ and CH designed the experiment. CJ ran the experiment and collected the data. CJ and CH analyzed the data and wrote the manuscript.

## Conflict of Interest Statement

The authors declare that the research was conducted in the absence of any commercial or financial relationships that could be construed as a potential conflict of interest.

## Footnotes

1 As the N2b and N2c are not relevant for the current experiment, these subcomponents will not be discussed in detail here (for a comprehensive review see Patel and Azzam, 2005; Folstein and van Petten, 2008).

2 http://audacity.sourceforge.net/

3 http://www.millisecond.com/download/library/Oddball/

4 http://sccn.ucsd.edu/eeglab/

5 http://openwetware.org/wiki/Mass_Univariate_ERP_Toolbox

6 More specifically, the null hypothesis of the permutation test is that positive differences between conditions could have just as likely been negative differences and vice-versa. Thus, the distribution of the null hypothesis is symmetric around a difference of 0.

7 http://www.uzh.ch/keyinst/loreta.htm
